# Adaptation and psychometric evaluation of the revised social constraints scale for partners of adults with cancer

**DOI:** 10.3389/fpsyg.2026.1784985

**Published:** 2026-05-07

**Authors:** Shuangshuang Zhang, Yingying Xu, Xin Xu

**Affiliations:** 1 Nursing Department, Sir Run Run Shaw Hospital, Zhejiang University School of Medicine, Hangzhou, China; 2 Department of Gastrointestinal Surgery, Nanjing Drum Tower Hospital the Affiliated Hospital of Nanjing University Medical School, Nanjing, China

**Keywords:** cancer, partners, scale adaptation, social constraints, validation

## Abstract

**Background:**

Partners of adults with cancer often experience social constraints that limit their ability to express cancer-related thoughts, feelings, and concerns to their loved ones. However, no validated instrument is currently available in Simplified Chinese to assess partner-specific social constraints. This study aimed to culturally adapt and evaluate the psychometric properties of the Simplified Chinese version of the Social Constraints Scale for partners of adults with cancer (SC-SCS-P).

**Methods:**

The scale was developed using a two-phase approach. In the first phase, items from the existing Simplified Chinese Social Constraints Scale (SCS) were contextually adapted to reflect partners' experiences, with reference to the original scale developed by Lepore. In the second phase, the psychometric properties of the adapted scale were evaluated, including content validity by a Delphi study with five experts, factor structure, convergent validity, and reliability. A convenience sample of 415 partners of adults with cancer was recruited from four hospitals in Hunan and Jiangsu provinces between June and September 2024.

**Results:**

Exploratory factor analysis supported a three-factor structure, including alienation, health concern minimization, and emotional concealment. The SC-SCS-P demonstrated good internal consistency and test-retest reliability. Higher levels of perceived social constraints were associated with greater fear of cancer recurrence and communication problems, and with lower psychological resilience, supporting the construct validity of the scale.

**Conclusion:**

The 15-item SC-SCS-P is a reliable and valid instrument for assessing partner-specific social constraints among partners of adults with cancer in China. This scale fills an important measurement gap and provides a foundation for future research and the development of communication-focused interventions, with implications for clinical practice and policy.

## 1 Introduction

Cancer is a major societal, public health, and economic problem nowadays, with approximately 20 million new cases of cancer emerged in 2022 alongside 9.7 million cancer deaths (Bray et al., 2024). In China, there were around 4.82 million new cancer cases and 2.57 million new deaths arising from cancer (Diao et al., 2025). Owing to the shift in cancer care from hospital- to home-based settings, family members are more frequently required to provide support to their adult relatives with cancer after discharge. Families play a crucial role in supporting cancer patients during periods of heightened vulnerability caused by illness. Partners, in particular, play a prominent role within the household context by providing daily care and emotional support to the individuals with cancer (Yoshimochi et al., 2018).

Social constraints are a major problem for adults with cancer and their caregivers. In the cancer context, social constraints represent negative social responses to the disclosure of cancer-related experiences, such as the ways others act, think or feel (Lepore and Revenson, 2007). When social constraints are perceived, adults with cancer tend to avoid thinking or talking about cancer-related thoughts and feelings, which can affect social and cognitive coping processes and influence their adjustment to cancer. Similar to adults with cancer, their partners may also experience social constraints. The literature documents that an unsupported social environment, such as myths, misconceptions and causal beliefs about cancer, can lead to gossip and stigmatization from society (Boamah Mensah et al., 2021; e Silva et al., 2010). Close relatives and acquaintances of adults with cancer may behave differently, sometimes feeling uncomfortable after being informed of the diagnosis (Nasiri et al., 2016). Partners may refrain from sharing concerns to avoid worrying family members, prevent conflict or protect the patients' feelings (Zahlis and Lewis, 2010). They often feel compelled to maintain strength to support the patients (Zahlis and Lewis, 2010). Moreover, some male partners may avoid discussing their experiences because they perceive it as inconsistent with their gender role, thus keep their thoughts private (Zahlis and Lewis, 2010; Hilton et al., 2000). They may also avoid disclosure because they do not want to burden others or feel that others would not understand their situation (Zahlis and Lewis, 2010). Consequently, partners may experience constraints in expressing their cancer-related thoughts, feelings or concerns to their loved ones living with cancer. This highlights the importance of measuring social constraints in partners of adults with cancer. Additionally, social constraints on disclosure may be heightened in Chinese cultural contexts, where norms emphasizing emotional restraint hinder emotional expression and contribute to reluctance to discuss personal concerns (Wang et al., 2020).

Social constraints are commonly measured using the social constraints scale (SCS), which exists in three versions having 5, 12, and 15 items, respectively (Lepore and Revenson, 2007). The 15-item version of the SCS is the most widely used, assessing the frequency of social constraints experienced by individuals with cancer. The scale was designed as unidimensional and these three versions were generated to tailor to different sources of social constraints, such as life-threatening illness, victimization or bereavement, with reference to particular stressors from a spouse, other family members or friends (Lepore and Revenson, 2007). The SCS has been applied across various cancer populations, demonstrating excellent reliability and validity (Lepore and Revenson, 2007; Koutrouli et al., 2016; Yeung et al., 2017; Luo et al., 2023). Cross-cultural adaptations show differing factor structures. The traditional Chinese version retains a unidimensional structure (Yeung et al., 2017), whereas the Simplified Chinese version identifies two factors (i.e., perceptual avoidance; reveal and conceal) (Luo et al., 2023). In contrast, the Greek version demonstrates a three-factor structure, including unsupported behaviors; avoidant behaviors; suggestions for distraction and pretense (Koutrouli et al., 2016).

The SCS has been adapted and used in US samples to assess social constraints of partners from patients with cancer; however, its psychometric properties were not reported (Cohee et al., 2017; Soriano et al., 2021). Moreover, no scale in the simplified Chinese language currently exists to assess partners' social constraints from adults with cancer. To address this gap, the present study aimed to culturally adapt and test the psychometric properties of the simplified Chinese version of the SCS for partners (SC-SCS-P), which was specifically designed to measure partner-specific social constraints from adults with cancer. A partner-specific, psychometrically robust tool will enable cancer research on communication barriers, their social patterning, and responsiveness to policy and practice interventions.

## 2 Method

This study employed a psychometric validation design comprising two phases. The first phase included scale adaptation and content validity. The second phase included a cross-sectional study to evaluate internal consistency, factor structure and construct validity and a longitudinal study to assess test-retest reliability.

### 2.1 Phase I scale adaptation and content validity

Phase I of scale adaptation and content validity included three components, including adaptation of the SCS to partners of adults with cancer, assessment of content validity by expert panels, and pilot testing of the adapted SCS to ensure conceptual clarity and contextual relevance.

#### 2.1.1 Adaptation of the SCS to the partners of adults with cancer

The adaptation followed the best-practice recommendation proposed by Heggestad et al. (2019). Formal permission to adapt the scale was obtained from the original developers. Because a validated Simplified Chinese version of the SCS already existed, the present study focused on perspective shift and contextual refinement rather than linguistic translation. Therefore, forward and back translation procedures were not required. The adaptation of the Social Constraints Scale (SCS) for partners of adults with cancer was conducted based on the 15-item Simplified Chinese SCS developed by Luo et al. (2023) and the original SCS items (Lepore and Revenson, 2007). The goal of the adaptation was to contextualize item wording so that items explicitly reflected partners' experiences in caregiving and family interactions. An initial draft of the partner-version items was prepared by a researcher with comprehensive research and clinical experience in partners' experience of adults with cancer. The adaptation involved contextual modifications to the items in the Simplified Chinese version by rephrasing statements to capture partners' experiences. To orient items toward the partners' perspectives, stems of most of the items were rephrased to target the patient–partner interaction; specifically, subject references were adjusted from “he/she (refers to the partner)” to “patient” where appropriate to reflect the vantage point of the respondent as the partner of an adult with cancer. This method has been used widely in scale revision and validation (Marín-Maicas et al., 2024; Efthymiou et al., 2019; Bian et al., 2018). In order to better capture partner-relevant phenomena, some items were subjected to item-level rewording.

#### 2.1.2 Content validity from expert panels

The adjustments to the scale were documented and retained for expert review. All changes preserved the original items' intent and scale structure to maintain comparability with prior SCS applications while improving contextual relevance for partners. The proposed scale was reviewed by a panel of five experts to establish content validity. Content validity refers to the extent to which a scale accurately reflects the concept it is intended to measure. In this study, content validity was evaluated at the item level using the I-CVI. For each item, the I-CVI was calculated as the proportion of experts assigning a relevance rating of 4 or 5 relative to the total number of participating experts (Polit et al., 2007). An I-CVI of ≥0.8 was predefined as the threshold for item inclusion. Experts were recruited from the research team's professional network and were eligible if they had more than 5 years of cancer-related research or clinical experience, held at least a master's degree and had published at least one paper in cancer research in a peer-reviewed journal. The panel comprised three experts in cancer nursing and research, one in clinical cancer nursing and one was a statistician with a research focus in cancer. Four of the expert are with PhD educational level and one with master educational level, with 10–27 years working experience. The proposed scale was sent to the experts at least 2 days prior to the individual meetings, which took place within 2 days in May 2024. The adapted scale was revised according to the comments provided by the experts and then sent out for further review. This iterative procedure was repeated until all the items in the scale have achieved an I-CVI ≥0.8.

#### 2.1.3 Pilot testing

To further assess comprehensibility, a pilot test was conducted with a sample of 12 partners of adults with cancer to evaluate the wording, expression and appropriateness of the items. Participants completed the draft scale and were probed using brief cognitive interview techniques to identify potential ambiguities or problematic phrasing. Modifications to the scale based on the partners' comments were made and the pilot-tested version of the scale was produced at this stage.

### 2.2 Phase II psychometric properties

Phase II employed a psychometric validation design comprising two components. The first component was a cross-sectional study to evaluate internal consistency, factor structure and convergent validity of the pilot-tested version of the SC-SCS-P. The second component was a longitudinal study designed to assess test–retest reliability.

#### 2.2.1 Participants

The inclusion criteria were as follows: (1) self-identified partners (defined as individuals in legally married or long-term committed intimate relationships) of adults diagnosed with cancer at any stage and receiving any form of treatment, including surgery, chemotherapy, radiotherapy or targeted therapy; (2) aged 18 years or older; (3) fully conscious and capable of completing questionnaires; and (4) provision of informed consent. Individuals with a diagnosed psychiatric disorder were excluded. Using convenience sampling, partners of adults with cancer were recruited from four tertiary A hospitals, three in Hunan Province and one in Jiangsu Province, in China between June and September 2024.

#### 2.2.2 Sample size

For the exploratory factor analysis (EFA), the recommended sample size is typically 3–20 times the number of items in the scale (Williams et al., 2010). Accordingly, a sample of approximately 150 participants (10 participants × 15 items) was planned for the EFA. For the confirmatory factor analysis (CFA), a general guideline suggests a minimum of 200 participants (Kyriazos, 2018). Accounting for an anticipated 10% rate of invalid questionnaires, the target sample size for recruitment was set at no fewer than 389 participants.

#### 2.2.3 Measures

##### 2.2.3.1 Social constraints

The newly revised Simplified Chinese version of the 15-item SC-SCS-P was used to assess social constraints for the target population. The participants rated their experiences of social constraints by using a 4-point Likert-type scale ranging from 1 (“never”) to 4 (“often”). Scores of the scale can range from 15 to 60, with higher scores indicating higher levels of social constraints.

##### 2.2.3.2 Psychological resilience

Psychological resilience refers to the mental processes and behaviors that strengthen personal resources and protect an individual from the potential negative effects of stressors (Fletcher and Sarkar, 2013). In this study, resilience was measured using the 10-Item Connor-Davidson Resilience Scale (CD-RISC-10) (Campbell-Sills and Stein, 2007; Connor and Davidson, 2003). The scale uses a 5-point Likert scale (0 = “never,” 1 = “rarely,” 2 = “sometimes,” 3 = “often,” and 4 = “always”) to solicit responses. Total scores of the CD-RISC-10 are calculated by summing the scores of all items, with higher scores indicating greater psychological resilience. The Chinese version of CD-RISC-10 has been widely used in China and demonstrates good reliability and validity (Wang et al., 2010). In the present study, the Cronbach's α coefficient was 0.882.

##### 2.2.3.3 Fear of cancer recurrence (FCR)

FCR in partners was assessed using the Fear of Progression Questionnaire-Short Form (FoP-Q-SF) (Mehnert et al., 2006). The FoP-Q-SF is a 12-item self-report instrument that provides a multidimensional evaluation of FCR. Items are rated on a 5-point Likert scale, ranging from 1 (“never”) to 5 (“very often”), with higher total scores indicating greater levels of FCR. A total score of 34 or above is considered indicative of clinically significant FCR (Wu, 2016). The Chinese version of the FoP-Q-SF has been validated, showing a Cronbach's α of 0.853 in partners (Wu, 2016). In the present study, the scale demonstrated good internal consistency, with a Cronbach's α of 0.855.

##### 2.2.3.4 Communication problem

Communication problems between partners and adults with cancer were measured using the 15-item Cancer-related Communication Problem Scale (CRCP) developed by Kornblith et al. (2006), which reported a Cronbach's α of 0.81 in a sample of male partners. Items are rated on a 3-point Likert scale (1 = “incorrect,” 2 = sometimes correct, 3 = correct), with higher total scores indicating greater communication problems within the couple. In previous studies on Chinese partners of cancer patients, the CRCP has demonstrated good internal consistency, with Cronbach's α ranging from 0.719 to 0.88 (Cheng et al., 2021; Yang et al., 2022). In the present study, the scale showed a Cronbach's α of 0.636.

In addition, demographic information, including age, gender, employment status and education level, and patients' demographic and disease-related information were obtained from the participants.

#### 2.2.4 Data collection procedure

Data were collected by trained data collectors. Partners were recruited via referral from adults with cancer at the participating hospitals. Upon presenting at the hospital, data collectors screened participants for eligibility and explained the study procedures. After providing informed consent, partners self-completed the questionnaires, with assistance provided by the data collectors if required. For participants who experienced difficulty reading the items, the data collectors read the items and response options aloud without prompting or influencing responses. For the test–retest survey, partners who had completed the baseline questionnaire were invited to complete the SC-SCS-P again within a 21-day interval.

#### 2.2.5 Statistical analysis

Questionnaires with more than 15% missing responses across total items were excluded from the analysis. Missing data at item level for the measures were substituted with the mean of the remaining filled items on the scale for each subject. All statistical analyses were conducted using IBM SPSS Statistics for Windows, Version 26.0 (IBM Corp., Armonk, NY, USA), with a two-sided *P*-value of <0.05 considered statistically significant. Demographic and disease-related characteristics of the partners were summarized using frequencies, means and standard deviations. Validity assessment included evaluation of factor structure and convergent validity, while reliability was assessed through internal consistency and test–retest reliability. The sample was randomly split into two subsamples in SPSS, with one subsample used for EFA and the other for CFA.

##### 2.2.5.1 Factor structure

The factor structure was examined in three steps. In Step 1, item reduction was performed based on item–total correlations using Pearson's *r*. This correlation, calculated between an individual item and the total test score, helps identify items that do not adequately reflect the underlying psychological construct and may therefore be removed or revised (Wolf, 1967). Items with *r* < 0.30 were excluded from further analysis (Tavakol and Wetzel, 2020).

In Step 2, EFAs were conducted on the items retained after Step 1. Principal axis factoring with direct oblique rotation was employed. The appropriateness of conducting EFA was assessed using the Kaiser–Meyer–Olkin (KMO) measure and Bartlett's test of sphericity. KMO values ≥0.60 were considered acceptable, while values ≥0.70 indicated good sampling adequacy. A statistically significant Bartlett's test (*P* < 0.05) suggested that the correlation matrix was not an identity matrix, thus justifying factor extraction (Suan et al., 2017). Factor retention was guided by four criteria: (i) eigenvalues >1, (ii) inspection of the scree plot, (iii) interpretability of the retained factors and (iv) factor loadings >0.30. Eigenvalues indicate the proportion of variance explained by each factor, with values above 1 considered stable. The scree plot displays the eigenvalues of the factors or principal components to aid in determining the number of factors to retain. Factor loadings represent the correlation between an item and a factor, with loadings over 0.30 generally interpreted as moderate associations (Tavakol and Wetzel, 2020). The EFA subsample was used for the analyses in Steps 1 and 2.

In Step 3, the factor structure obtained from the EFA was further confirmed using CFA. The CFA was performed on the covariance matrix of the items using the CFA subsample. Maximum likelihood estimation was employed for model estimation and covariance indices. The adequacy of model fit was assessed using multiple fit indices (Hooper et al., 2008). The root mean square error of approximation (RMSEA) is an absolute fit index that evaluates the discrepancy between the model and the data per degree of freedom. RMSEA values between 0.05 and 0.08, together with a 90% confidence interval (CI) with a lower bound of 0.05 and an upper bound of 0.10, are considered acceptable, whereas values of <0.06 are considered excellent (Hu and Bentler, 2009; MacCallum et al., 1996). The comparative fit index (CFI) reflects the relative fit of the specified model compared with the null model; CFI values >0.95 indicate good fit, whereas values of >0.90 indicate acceptable fit (Hu and Bentler, 2009). Although a non-significant chi-square test (*P* > 0.05) suggests good model fit (Barrett, 2007), chi-square values are often statistically significant in large samples (Hooper et al., 2008). Therefore, the chi-square test results were reported for descriptive purposes only and were not used as a primary criterion for evaluating model fit.

Model modifications were guided by the modification indices (MIs), which identify potential improvements in model fit by suggesting additional paths or constraints that can be added. The interpretation of MI values was based on statistical significance and practical relevance. An MI value greater than 3.84 indicates that releasing the constraint (e.g., adding a path or covariance) would result in a significant improvement in model fit at *P* < 0.05 and should be considered for modification. An MI value exceeding 6.63 indicates a significant improvement at *P* < 0.01 and warrants strong consideration for modification. An MI value greater than 10.83 reflects a highly significant improvement at *P* < 0.001 and should be prioritized in model modifications (Just Enough, n.d.). However, only paths that are theoretically plausible would be considered for model modifications.

##### 2.2.5.2 Reliability

Internal consistency was evaluated using Cronbach's α across the entire sample. A Cronbach's α ≥0.70 was considered indicative of good internal reliability (Hendriks et al., 2013). Values at or above this threshold suggest that the items are sufficiently consistent, confirming that the scale is reliable. Test–retest reliability was assessed using intra-class correlation coefficients (ICCs) over a 21-day interval. An ICC of >0.75 indicates good test–retest reliability, while values between 0.50 and 0.75 indicate moderate reliability (Koo and Li, 2016).

##### 2.2.5.3 Floor and ceiling effects

The domains of the SC-SCS-P were examined for floor and ceiling effects and defined as the proportions of respondents achieving the minimum or maximum possible scores on the scale. Floor or ceiling effects were considered present if more than 30% of the total sample obtained the lowest or highest possible score, respectively (Kane, 2006).

##### 2.2.5.4 Convergent validity

Convergent validity was assessed using correlation analysis on the entire sample. Pearson's correlation was applied for variables with a normal distribution, while Spearman's correlation was used for variables that were non-normally distributed. Variables were considered non-normally distributed if the absolute value of skewness exceeded 2 or the absolute value of kurtosis exceeded 7 (Kim, 2013). We hypothesized that social constraints in partners would be negatively correlated with psychological resilience and positively correlated with FCR and communication problems. In addition, convergent validity was further supported by acceptable values of average variance extracted (AVE > 0.50) and composite reliability (CR > 0.70).

##### 2.2.5.5 Discriminant validity

Discriminant validity was evaluated using the Fornell–Larcker criterion and examination of inter-factor correlations. Discriminant validity was considered adequate when the square root of AVE exceeded inter-factor correlations and when correlations between constructs were below 0.75 (Cheung et al., 2024).

##### 2.2.5.6 Homogeneity testing and effect size

To examine differences in partners' social constraints across demographic characteristics, bivariate analyses were performed. Pearson's correlation was used for continuous variables (age and time since diagnosis), independent *t*-tests for binary variables (gender, employment status, surgery, chemotherapy, radiotherapy, and targeted therapy), and one-way ANOVA for categorical variables (educational level, cancer type, and cancer stage). When ANOVA results were significant, Bonferroni-adjusted *post-hoc* pairwise comparisons were conducted to control for multiple comparisons. Effect sizes were reported as Pearson's *r* for correlations, Cohen's *d* for independent-samples *t*-tests, and partial eta squared (partial η^2^) for one-way ANOVA.

#### 2.2.6 Ethical considerations

This study was approved by the Ethics Committee of the participating hospital (Approval ID: 20240101) and conducted with the cooperation of the nursing department managers at each site. All participants provided written informed consent prior to enrolment.

## 3 Results

### 3.1 Phase I scale adaptation and content validity

#### 3.1.1 Results of the scale adaptation

The primary modification involved changing the referent from “he/she (partner)” to “the patient.” Four types of adaptation were identified. First, for most items (Items 1, 3, 4, 5, 8, 13, 14, and 15), only referent modification was required. Second, Items 10–12 required additional adjustment of health-related pronouns (e.g., changing “your disease/health” to “his/her disease/health”) to maintain contextual alignment. Third, Item 6 involved illness ownership reattribution, in which “your own illness” was revised to “the patient's illness” to ensure contextual consistency. Fourth, Items 2 and 7 were rephrased to reflect perception-based wording (e.g., adding “You feel that”). Additionally, Item 2 involved content refinement by replacing “health condition” with “concern/feeling” to better capture emotional experience. Finally, Item 9 required clarification of the illness-related focus by changing “concerns about you” to “concerns about his/her own illness.” Detailed adaptation was shown in Table 1.

**Table 1:** Detailed adaptation.

Item number	SCS from Luo et al. (2023)	Adapted SC-SCS-P	Detailed adaptation
1.	When you try to talk with *him/her* about *your* disease, he/she changes the topic	When you try to talk to *the patients* about *his/her disease*, he/she changes the topic	Changing the referent from “he/she (partner)” to “the patient”
3.	You feel that *he/she* avoids being with you	You feel that *the patient* avoids being with you	
4.	When you bring up concerns or ideas related to health, *he/she* ignores or downplays them	When you bring up concerns or ideas related to health, *the patient* ignores or downplays them	
5.	You feel that *he/she* hides his/her emotions or feelings from you	You feel that *the patient* hides his/her emotions or feelings from you	
8.	When you try to talk to *he/she, he/she* only complains about his/her own problems	When you try to talk to the *patient, the patient* only complains about his/her own problems	
13.	You feel you have to hide your feelings related to the disease because your feeling makes *him/her* uncomfortable	You feel you have to hide your feelings related to the disease because your feeling makes *the patient* uncomfortable	
14	You feel you have to hide your feelings related to the disease because your feeling make *him/her* unpleasant	You feel you have to hide your feelings related to the disease because your feeling makes *the patient* unpleasant	
15.	You feel unhappy because *he/she* doesn't care/love you as you expected	You feel unhappy because *the patient* doesn't care/love you as you expected	
10.	*He/she* tells you not to worry so much about *your own* health	*The patient* tells you not to worry so much about *his/her* health	1. Changing the subject from “him/her (refers to the partner)” to “patient” 2. Changing health-related pronouns from “your” to “*his/her*”
11.	*He/she* asks you to think less about things related to *your* disease	*The patient* asks you to think less about things related to *his/her* disease
12.	You feel that *he/she* doesn't want to hear things related to *your* disease	You feel that *the patient* doesn't want to hear things related to *his/her* disease
6.	When you mention *your own illness, he/she* appears uncomfortable	When you mention *patient's* illness, *the patient* appears uncomfortable	1. Changing the subject from “he/she (refers to the partner)” to “patient” 2. Changing “your own illness” to “the patient's illness”
2.	He/she does not understand your health condition	You feel that the patient does not understand your concern/feeling	1. Changing the referent from “he/she (partner)” to “the patient” 2. Changing “health condition” to “concern/feeling” to better reflect emotional experience 3. Rephrasing to reflect perception-based wording
7.	*He/she* makes you think your problem is unimportant	You feel that *the patient* thinks your problem is unimportant	1. Changing the referent from “he/she (partner)” to “the patient” 2. Rephrasing to reflect perception-based wording
9.	*He/she* appears cheerful in your presence to hide his/her true feelings or concerns about *you*	*The patient* appears cheerful in your presence to hide his/her true feelings or concerns about his/her *own illness*	1. Changing the referent from “he/she (partner)” to “the patient” 2. Changing “concerns about you” to “concerns about his/her own illness”

#### 3.1.2 Result of the content validity from expert panels

The expert panel confirmed that the language used in the revised scale was appropriate, ensuring that the items were easily understandable and relevant to the target population. One recommendation was implemented: in Item 2, the word “situation” was replaced with “feeling/concern.” The I-CVI for all items in this version of the scale was 1.0.

#### 3.1.3 Pilot testing

Twelve partners of adults with cancer were invited to participate in the pilot testing. No additional modifications were required following this pilot phase. Consequently, the pilot-tested SC-SCS-P was generated for subsequent psychometric testing.

### 3.2 Phase II psychometric properties

#### 3.2.1 Sociodemographic characteristics

A total of 415 questionnaires were distributed to partners of adults with cancer, of which 401 were with missing data less than 15% and returned as valid, yielding a response rate of 96.63%. The mean age of the partners was 48.46 years (SD = 9.41), ranging from 25 to 72 years. Nearly two-thirds were male (63.1%), the majority completed junior secondary school (31.9%) or high secondary school or college (31.4%) and 49.9% were unemployed. Regarding the patients' characteristics, the mean age was 48.16 years (SD = 9.84), ranging from 23 to 74 years. Nearly two-thirds were female (63.1%), 25.9% were diagnosed with breast cancer and 28.4% were in stage IV. Among the 401 patients, 41.4% had undergone surgery, 51.4% received chemotherapy, 23 underwent radiotherapy and 92 received targeted therapy. Further details are presented in Table 2.

**Table 2:** Sociodemographic characteristics of the partners (*n* = 401).

Population	Variable	Group	*n*	(%)
Partners	Age	25–72 (48.46 ± 9.406)	336	83.8
	Gender	Female	147	36.7
		Male	253	63.1
		Unclear	1	0.2
	Education level	Primary school or below	41	10.2
		Junior secondary school	128	31.9
		High secondary school or college	126	31.4
		University or above	63	15.7
		Unclear	43	10.7
	Employment status	Unemployed	200	49.9
		Employed	155	38.7
		Unclear	46	11.5
Patients	Age	23–74 (48.16 ± 9.835)	397	99.0
	Gender	Female	253	63.1
		Male	147	36.7
		Unclear	1	0.2
	Time since diagnosis (months)	0–195 (8.01 ± 22.53)	/	/
	Cancer type	Breast cancer	104	25.9
		Thyroid cancer	40	10
		Nasopharyngeal carcinoma	39	9.7
		Colorectal cancer	39	9.7
		Lung cancer	38	9.5
		Oral cancer	37	9.2
		Gynecological cancers	36	9
		Urological cancers	11	2.7
		Neurological cancers	10	2.5
		Liver cancer	9	2.2
		Osteosarcoma	9	2.2
		Gastric cancer	9	2.2
		Others (including head and neck cancers, pancreatic cancer, esophageal cancer, etc.)	20	4.99
	Stage	0	12	3.0
		1	82	20.4
		2	68	17.0
		3	91	22.7
		4	114	28.4
		Unclear	34	8.5
	Surgery	No	227	56.6
		Yes	166	41.4
		Unclear	8	2.0
	Chemotherapy	No	187	46.6
		Yes	206	51.4
		Unclear	8	2.0
	Radiotherapy	No	370	92.3
		Yes	23	5.7
		Unclear	8	2.0
	Targeted therapy	No	301	75.1
		Yes	92	22.9
		Unclear	8	2.0

#### 3.2.2 Item-total correlation

Table 3 shows the item–total correlations of all the 15 items in the scale, ranging from 0.394 to 0.667. Thus, all items were retained in the scale for EFA. Although Item 2 demonstrated a slightly lower correlation (*r* = 0.394), it was retained because its removal did not improve internal consistency. Specifically, Cronbach's alpha remained unchanged (α = 0.841) when Item 2 was deleted. Therefore, Item 2 was considered to contribute meaningfully to the overall construct. Furthermore, Item 2 captures the core perception of being emotionally misunderstood, which is conceptually central to social constraints.

**Table 3:** Item–total correlations of the scale.

Number	Items	Item-total correlation
1	When you try to talk to the patients about his/her disease, he/she changes the topic	0.581
2	You feel that the patient does not understand your situation	0.394
3	You feel that the patient avoids being with you	0.473
4	When you bring up concerns or ideas related to health, the patient ignores or downplays them	0.566
5	You feel that the patient hides his/her emotions or feelings from you	0.667
6	When you mention patient's illness, the patient appears uncomfortable	0.589
7	You feel that the patient thinks your problem is unimportant	0.569
8	When you try to talk to the patient, the patient only complains about his/her own problems	0.569
9	The patient appears cheerful in your presence to hide his/her true feelings or concerns about his/her own illness	0.578
10	The patient tells you not to worry so much about his/her health	0.447
11	The patient asks you to think less things related to his/her disease	0.552
12	You feel that the patient doesn't want to hear things related to his/her disease	0.654
13	You feel you have to hide your feelings related to the disease because your feeling makes the patient uncomfortable	0.590
14	You feel you have to hide your feelings related to the disease because your feeling makes the patient unpleasant	0.602
15	You feel unhappy because the patient doesn't care/love you as you expected	0.509

#### 3.2.3 Exploratory factor analysis

The total sample was randomly split into two subsamples: an EFA-subsample (*n* = 200) and a CFA-subsample (*n* = 201). Table 4 presents the EFA results. For the EFA-subsample, the KMO measure of sampling adequacy was 0.807, and Bartlett's test of sphericity yielded and χ^2^ value 912.78 and *P*-value of <0.0001, indicating that the data were suitable for factor analysis.

**Table 4:** Results of exploratory factor analysis (EFA; *n* = 200).

Item number	Intended dimensions	Factor 1	Factor 2	Factor 3	Communalities
Item 1	Alienation	**0.431**	0.066	0.045	0.236
Item 2		**0.333**	−0.022	0.036	0.118
Item 3		**0.520**	−0.033	−0.103	0.218
Item 4		**0.559**	−0.031	0.011	0.305
Item 5		**0.437**	0.195	0.190	0.439
Item 6		**0.505**	−0.028	0.130	0.324
Item 7		**0.698**	−0.088	−0.022	0.433
Item 8		**0.511**	0.161	0.026	0.369
Item 9		**0.353**	0.295	0.073	0.339
Item 12		**0.427**	0.274	0.104	0.423
Item 15		**0.392**	0.006	0.001	0.156
Item 10	Health concern minimization	−0.088	**0.873**	−0.036	0.693
Item 11		0.080	**0.717**	0.023	0.579
Item 13	Emotional concealment	0.006	−0.115	**0.983**	0.908
Item 14		0.012	0.076	**0.782**	0.668

Boldface indicates that the item loads on that factor.

**Figure 1:**
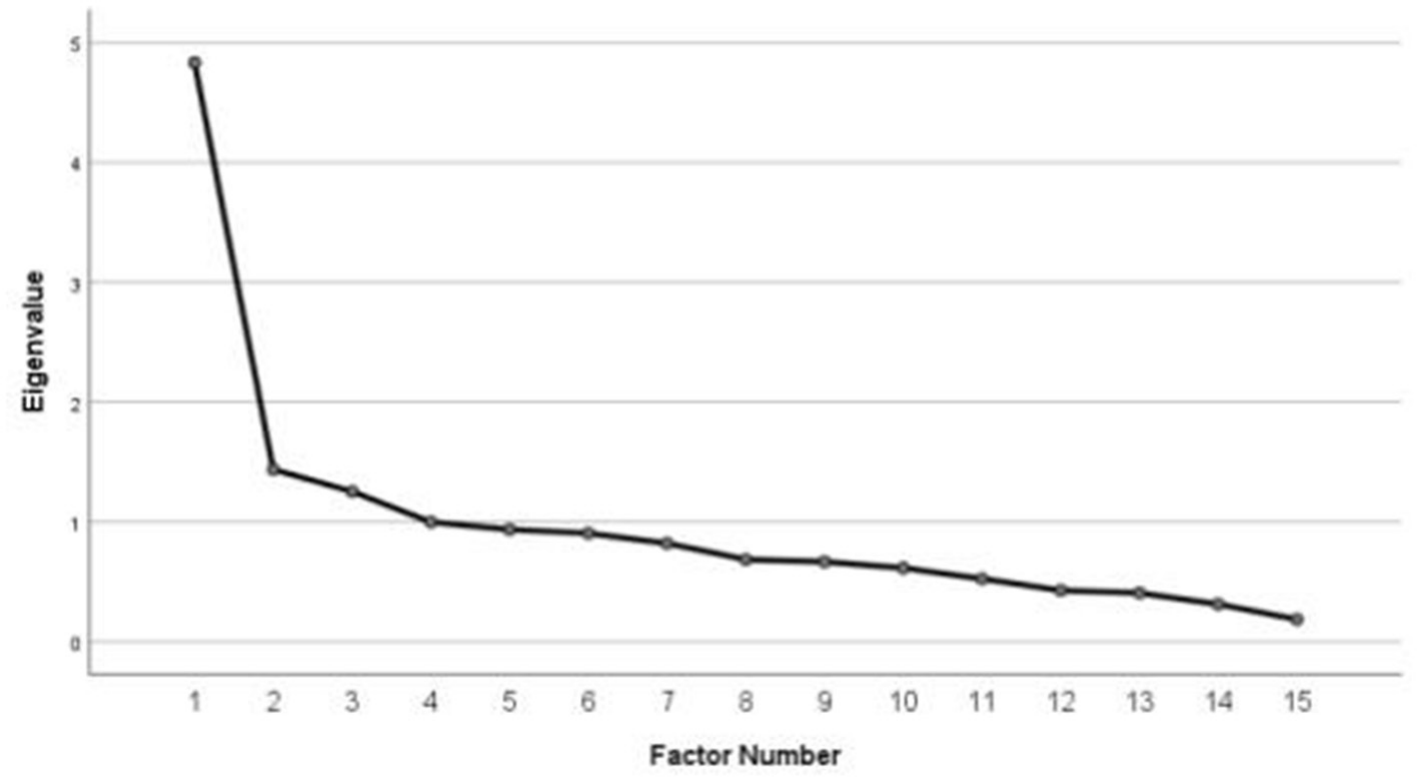
Screen plot.

A three-factor solution was retained based on the four factor-retention criteria. Three factors had eigenvalues greater than 1.0 (4.833, 1.437, and 1.253) and inspection of the scree plot revealed a clear leveling-off after the third factor (Figure 1). All items had factor loadings >0.3, and no item loaded significantly on more than one factor; therefore, all 15 items were retained (Table 4). Communalities ranged from 0.12 to 0.91, with 11 items exceeding 0.30, indicating that most items shared adequate variance with the extracted factors. Although the communality of four items (Item 1, 2, 3, and 15) were lower than, their factor loading exceeded 0.30 and it did not demonstrate problematic cross-loading; therefore, it was retained to preserve content coverage. A three-factor solution was supported, which accounted for 50.16% of the total variance. Specifically, Factor 1 explained 32.22%, Factor 2 explained 9.58%, and Factor 3 explained 8.36% of the variance. Unlike the original unidimensional structure, the SC-SCS-P demonstrated a three-factor structure. Factor 1 covers 11 items of “Alienation,” factor 2 has two items in “Minimization of health concern” and factor 3 has two items in “Emotional concealment.” Although Factors 2 and 3 consist of two items each, both demonstrated strong loadings (>0.70), supporting structural stability.

As shown in Table 5, the factor correlation matrix following oblique rotation showed moderate inter-factor correlations, ranging from 0.338 to 0.496, indicating that the three dimensions were related yet distinct.

**Table 5:** Factor correlation matrix.

Factors	Factor 1	Factor 2	Factor 3
Factor 1	/	/	/
Factor 2	0.396	**/**	/
Factor 3	0.489	0.338	/

#### 3.2.4 Confirmatory factor analysis

To evaluate the appropriateness of both the original single-factor structure and the three-factor structure obtained from EFA, CFA were conducted using the maximum likelihood method. Two models were tested: a single-factor model (Model 1) and the three-factor model derived from EFA (Model 2). The results are presented in Table 6. The single-factor model (Model 1) showed a poor model fit to the data [χ^2^ = 367.089, df = 90, RMSEA = 0.124 (90% CI: 0.111–0.137), CFI = 0.667]. The three-factor model (Model 2) has model fit indexes that are close to their cut-off points [χ^2^ = 173.568, df = 87, RMSEA = 0.071 (90% CI: 0.055–0.086), CFI = 0.896]. The MI results indicated that adding a path from Factor 2 to Item 9 would yield the highest MI value (21.691); however, this modification was not implemented because the content of Item 9 was deemed only weakly relevant to Factor 2 (Minimization of Health Concern).

**Table 6:** Fit statistics.

Model	χ^2^	df	*P*-value	RMSEA (90% CI)	CFI
Model 1–Original one factor model	367.089	90	<0.0001	0.124 (0.111–0.137)	0.667
Model 2–Three-factor model from EFA	173.568	87	<0.0001	0.071 (0.055–0.086)	0.896
Model 3–Model 2 plus correlation between Items 6 and 15	155.162	86	<0.0001	0.063 (0.047–0.079)	0.917

**Figure 2:**
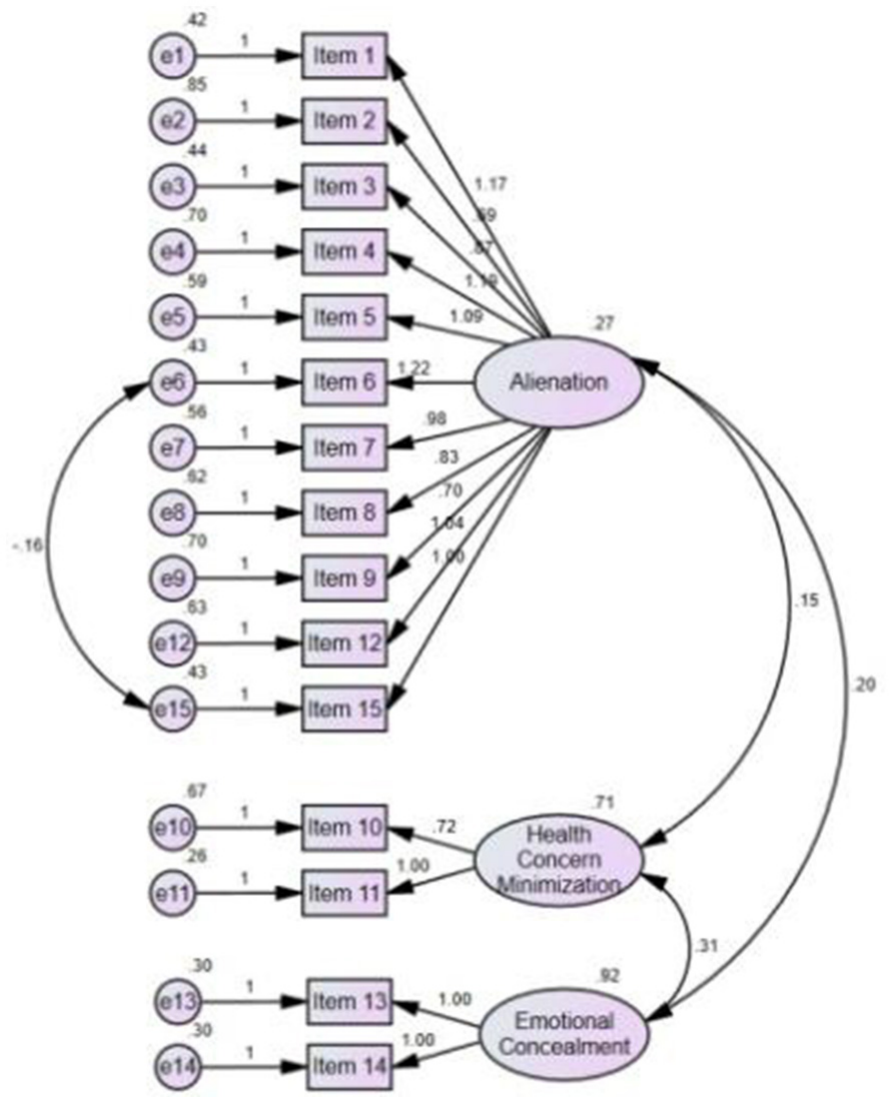
Final three-factor model indicated by CFA (*n* = 201).

Further examination of the MI results indicated that model fit can be improved by allowing the residuals of Items 6 and 15 in Factor 1 to correlate (MI = 14.752). The model was rerun with this adjustment, producing a respecified model (Model 3) with acceptable fit indices: χ^2^ = 155.162, df = 86, RMSEA = 0.063 (90% CI: 0.047–0.079) and CFI = 0.917 (Table 6). No additional modifications were made. The final three-factor model is illustrated in Figure 2.

#### 3.2.5 Reliability of the scale

Table 7 presents the number of items, internal consistency, test-retest reliability and ceiling and floor effects of the SC-SCS-P. The overall Cronbach's α was 0.841, indicating good internal consistency. The values for Alienation subscale was 0.817. For the two-item Health Concern Minimization subscale, the inter-item correlation was 0.562 (*P* < 0.001), yielding a Spearman–Brown coefficient of 0.719. For the two-item Emotional Concealment subscale, the inter-item correlation was 0.759 (*P* < 0.001), yielding a Spearman–Brown coefficient of 0.863. These values indicate acceptable to good internal consistency for two-item measures. A total of 33 partners completed the SC-SCS-P again within 21 days. Test–retest reliability was strong for the overall scale and the “Alienation” and “Emotional Concealment” subscales (ICCs > 0.80), whereas the “Minimization of Health Concern” subscale demonstrated moderate reliability (ICC = 0.643). Ceiling and floor effects were examined for the total scale and all three subscales. Across the overall scale and subscales, only 0.2%−11.2% of respondents achieved the highest possible score (<30%), indicating no ceiling effect. For the floor effect, 2.5%−26.2% of participants obtained the lowest possible score, showing no floor effect.

**Table 7:** Internal consistency, test–retest reliability, ceiling and floor effects of the scale.

Scale/dimensions	Number of items	Cronbach's α	ICC (95% CI)	Ceiling effect *n* (%)	Floor effect *n* (%)
Overall	15	0.841	0.90 (0.80, 0.95)	1 (0.2%)	10 (2.5%)
Alienation	11	0.817	0.846 (0.71, 0.93)	1 (0.2%)	17 (4.2%)
Health concern minimization	2	0.719	0.643 (0.37, 0.81)	24 (6.0%)	70 (17.5%)
Emotional concealment	2	0.863	0.808 (0.65, 0.91)	45 (11.2%)	105 (26.2%)

ICC, intra-class correlation coefficient.

#### 3.2.6 Convergent validity

All variables met the criteria for normality, and Pearson correlations (*r*) were computed. Skewness ranged from −0.471 to 0.411, and kurtosis ranged from −1.110 to 0.687, indicating acceptable normality (|skewness| <2; |kurtosis| <7). As shown in Table 8, the total SC-SCS-P score and its three subscales were significantly and negatively correlated with psychological resilience and positively correlated with FCR and communication problems, supporting convergent validity. The only exception was the “Minimization of Health Concern” subscale, which did not show a significant correlation with psychological resilience. These results indicate that higher social constraints are associated with lower resilience and higher FCR and communication difficulties, consistent with theoretical expectations.

**Table 8:** Correlations of the overall and three domain scores of SC-SCS-P with psychological resilience, FCR and communication problem in partners of adults with cancer.

Social constraints	Psychological resilience		FCR		Communication problem	
	Correlation	*P*-value	Correlation	*P*-value	Correlation	*P*-value
Overall	−0.182	<0.001	0.338	<0.001	0.139	0.005
Dimensions						
Alienation	−0.208	<0.001	0.283	<0.001	0.111	0.026
Health concern minimization	0.032	0.512	0.222	<0.001	0.111	0.026
Emotional concealment	−0.125	0.012	0.292	<0.001	0.117	0.019

#### 3.2.7 Discriminant validity of the SC-SCS-P

As shown in Table 9, the CR values ranged from 0.68 to 0.86, indicating acceptable internal consistency. The AVE values ranged from 0.32 to 0.75. Although the AVE for Alienation was slightly below the recommended threshold of 0.50, its CR was satisfactory. The square roots of AVE for all constructs exceeded the corresponding inter-factor correlations, and inter-factor correlations were below 0.75, supporting adequate discriminant validity.

**Table 9:** Discriminant validity of the SC-SCS-P.

Construct	AVE	CR	√AVE	Alienation	Health concern	Emotional concealment
Alienation	0.318	0.83	0.564	/	0.341	0.403
Health concern minimization	0.522	0.68	0.723	0.341	/	0.364
Emotional concealment	0.751	0.86	0.866	0.403	0.364	/

#### 3.2.8 Level of social constraints in partners and correlations with demographic variables

Table 10 shows the level of social constraints in partners and its correlations with demographic variables. According to the homogeneity test, education level demonstrated a significant difference in the level of social constraints in partners (*P* = 0.003, partial η^2^ = 0.039). Bonferroni-adjusted *post-hoc* pairwise comparisons indicated that partners with primary school education or below reported significantly higher social constraints than those with high secondary school/college (*P* = 0.002) and university or above (*P* = 0.034). No other pairwise differences were statistically significant after adjustment. Variables of age, gender and employment status in partners, and variables of age, gender, time since diagnosis, cancer type, stage, surgery, chemotherapy, radiotherapy and targeted therapy in adults with cancer showed non-significant results (*P* > 0.05) and small effect size.

**Table 10:** Social constraints level in partners of adults with cancer by demographic variables.

Population	Variable	Group	*n*	Social constraints (Mean ±SD)	*P*-value	Effect size
Characteristic in partners	Age	/	397	30.22 ± 7.93	0.792	0.013^a^
	Gender	Female	253	29.79 ± 7.89	0.179	−0.140^b^
		Male	147	30.89 ± 7.96		
	Education level	Primary school or below	41	**33.44** **±8.64**	0.003^*^	0.039^c^
		Junior secondary school	128	30.35 ± 6.97		
		High secondary school or college	126	28.47 ± 7.97		
		University or above	63	29.17 ± 7.59		
	Employment status	Unemployed	200	30.54 ± 7.54	0.071	0.016 ^b^
		Employed	155	29.05 ± 7.92		
Characteristic in adults with cancer	Age	/	336	30.22 ± 7.93	0.443	0.042
	Time since diagnosis	/	388	30.22 ± 7.93	0.077	0.090
	Cancer type	Breast cancer	104	29.54 ± 8.18	0.852	0.024^c^
		Thyroid cancer	40	28.98 ± 7.14		
		Nasopharyngeal carcinoma	39	30.72 ± 7.42		
		Colorectal cancer	39	30.15 ± 7.87		
		Lung cancer	38	31.55 ± 7.38		
		Oral cancer	37	31.65 ± 8.80		
		Gynecological cancers	36	30.67 ± 9.13		
		Urological cancers	11	27.45 ± 7.65		
		Neurological cancers	10	29.40 ± 10.02		
		Liver cancer	9	31.67 ± 7.19		
		Osteosarcoma	9	28.89 ± 6.70		
		Gastric cancer	9	29.33 ± 7.81		
		Others (including head and neck cancers, pancreatic cancer, esophageal cancer, etc.)	20	31.75 ± 6.97		
	Stage	0	12	29.25 ± 7.81	0.742	0.005 ^c^
		1	82	30.44 ± 8.08		
		2	68	29.41 ± 7.77		
		3	91	30.12 ± 7.84		
		4	114	30.96 ± 7.82		
	Surgery	No	227	30.34 ± 7.61	0.824	0.023 ^b^
		Yes	166	30.16 ± 8.39		
	Chemotherapy	No	187	30.32 ± 8.26	0.909	0.012 ^b^
		Yes	206	30.22 ± 7.65		
	Radiotherapy	No	370	30.17 ± 8.00	0.346	−0.203 ^b^
		Yes	23	31.78 ± 6.83		
	Targeted therapy	No	301	30.32 **±** 7.95	0.815	0.028^b^
		Yes	92	30.10 ± 7.94		

^a^Pearson's r.

^b^Cohen's d.

^c^Partial η^2^.

^*^P < 0.05.

Bold values indicate groups showing statistically significant differences in the one-way ANOVA.

## 4 Discussion

We have culturally adapted and tested the psychometric properties of the Simplified Chinese version of SCS for partners (SC-SCS-P), which measures partner-specific social constraints from adults with cancer. The scale demonstrated excellent convergent validity, test-retest reliability and internal consistency and a 3-factor factor structure, indicating that the 15-item SC-SCS-P is a valid and reliable measurement tool for assessing a partner's social constraints from adults with cancer. Consistent with prior literature, partners across collectivist (e.g., Iran) and individualist contexts (e.g., Canada, the UK and the USA) frequently report feeling constrained in expressing their thoughts, emotions or concerns to loved ones with cancer (Boamah Mensah et al., 2021; e Silva et al., 2010; Nasiri et al., 2016; Bu et al., 2025a; Gutierrez et al., 2016; Traboulssi et al., 2022; Zahlis and Shands, 1991). Thus, assessing social constraints in the partners of cancer patients is essential. Therefore, this scale can serve as a unique instrument for evaluating such constraints in partners of adults with cancer in China and in different countries.

Factor analysis indicated that the SC-SCS-P with a three-factor solution was optimal, explained 50.16% of the variance. All the 15 items were retained, with eleven items in the factor of alienation, two items in the factor of health concern minimization and two items in emotional concealment. The first factor of alienation refers to a sense of disconnection or estrangement from oneself, others or society. It can manifest emotionally, socially or existentially. The second factor of health concern minimization refers to downplaying or dismissing health issues, either by individuals (e.g., ignoring symptoms) or institutions (e.g., healthcare providers underestimating patient complaints). The third factor of emotional concealment refers to hiding or suppressing emotions to conform to social norms, avoid conflict or protect privacy. The three dimensions align with the three sources of social constraints identified by Lepore et al. (1996), including (1) inappropriate or insensitive behaviors from the available social network, (2) absence of supportive people to talk and (3) outright negative reactions of others. Factor analysis of the SC-SCS-P indicated three types of social constraints, namely, alienation, health concern minimization and emotional concealment. The first dimension of alienation, encompassing items, such as patients changing the topic when discussing their illness (Item 1), appearing not to understand their partners' feelings (Item 2), avoidance (Item 3), ignoring or minimizing the partners' feelings (Item 4), hiding their own feelings (Item 5) and showing discomfort in discussing the illness (Items 6, 7, 8, 9, 12, and 15), reflects social constraints stemming from inappropriate or unsupportive social behaviors. These constraints may cause feelings of misunderstanding, dismissal and emotional isolation. The second dimension of health concern minimization (Items 10 and 11), characterized by patients' downplaying their health concerns or discouraging caregivers from worrying excessively, corresponds to social constraints related to avoidance or denial, limiting open communication about trauma-related thoughts and feelings. The third dimension of emotional concealment (Items 13 and 14), where partners feel the need to hide their emotions about the illness to avoid causing discomfort or unhappiness in patients, reflects the impact of negative social reactions and avoidance within the support network. Further, emotional concealment dimension may also be understood within the broader Chinese sociocultural context. In Chinese culture, maintaining interpersonal harmony and preserving “face” are often emphasized in close relationships. Individuals may therefore regulate emotional expression to avoid causing distress, burden, or relational tension within the family (Ho, 1976).

We have replicated the three-factor solution from EFA in the CFA by using an independent random subsample. Acceptable model fit was obtained after a *post hoc* modeling adjustment with the addition of the correlation between the residuals of the Items 6 and 15. The potential reason for the negative correlation between Items 6 and 15 lies in how the health-related stress in adults with cancer impacts relational dynamics, creating emotional distance and perceived neglect. Item 6 of “When you mention patient's illness, the patient appears uncomfortable” describes partners' perception of the discomfort with discussing cancer from the adult with cancer. This reaction may stem from anxiety, denial, vulnerability and cultural stigma. The patient may withdraw emotionally as a coping mechanism to avoid confronting distressing emotions or to maintain psychological defense, which is very likely in the context of the Chinese culture (Zhang et al., 2024). Item 15 “You feel unhappy because the adult with cancer doesn't seem to care about you as much as you expected.” describes partners' perception of reduced love or care. The negative correlation between Items 6 and 15 may reflect that in some cases, partners interpret the patients' discomfort in discussing cancer (Item 6) not as emotional neglect but as an attempt to protect them from distress (i.e., protective buffering). In the Chinese cultural context, where indirect expression of care is common, such avoidance can be perceived as a form of concern, thereby reducing the feeling of being uncared for (Item 15).

The SC-SCS-P and its three domains exhibited good internal consistency and test–retest reliability, with a range between 0.719 and 0.863. No ceiling or floor effects were observed for the overall scale. Good test–retest reliability of the proposed scale was observed, but the reliability in the health concern minimization subscale was moderate. The moderate test–retest reliability in the health concern minimization subscale may reflect the inherently variable nature of its content. This subscale consists of two items: “The patient tells you not to worry so much about his/her health” and “The patient asks you to think less about things related to his/her disease.” These items capture interpersonal expressions of reassurance or downplaying health concerns. Such behaviors are likely to fluctuate over time depending on the patient's current health condition, emotional state or recent experiences, leading to temporal variability higher than that of stable constructs. In addition, the limited number of items in this dimension may have further reduced the stability of the estimate. Amongst the existing scales used to assess social constraints in adults with cancer, only one study reported test–retest reliability of the scale, which was 0.826 (Luo et al., 2023). The findings of the current study have extended the evidence in reliability of the scale from the patient population to the partner population. Although the AVE for the Alienation dimension was slightly below the recommended threshold of 0.50, its composite reliability was satisfactory and discriminant validity was supported. The relatively lower AVE may reflect the broader conceptual scope of alienation in the partner context, which encompasses multiple interpersonal experiences and perceptions.

The current findings of the correlational analysis were strongly supported by the integrative model of FCR developed by Simonelli et al. (2017) and hence support the convergent validity of the scale. As postulated by the integrative model, significant results were found, in which social constraints negatively correlated with psychological resilience, and positively correlated with FCR and communication problem were identified in the overall scale and all the three dimensions. Although social constraints were associated with communication problems, the correlations were low (*r* = 0.139), indicating that social constraints represent perceived interpersonal restriction rather than general communication quality. Unlike communication problems, which reflect frequency or effectiveness of communication, social constraints capture perceived discouragement or inhibition in illness-related discussions. This supports the conceptual distinction between these constructs. However, the negative correlation between health concern minimization and psychological resilience was non-significant. Partners with strong psychological resilience and reduced communication problems tended to have a low level of social constraint. Social constraints reflect behaviors, cognitions and feelings and the individual's construal of them (Lepore and Revenson, 2007). Resilience denotes positive adaptation or the capacity to sustain or regain mental health in the face of adversity (Herrman et al., 2011). Therefore, partners with strong psychological resilience are likely to maintain mental conditions regardless of unfavorable social constraints. The non-significant correlation between health concern minimization and psychological resilience is theoretically reasonable because the factor “health concern minimization” reflects partners' perceptions that patients downplay their own health concerns or discourage their loved ones from worrying excessively, which is partners' perception of the behaviors from the adults with cancer, and may thus not relate to partners' psychological resilience. In contrast, psychological resilience is the individual's ability to adapt positively and maintain psychological functioning in the face of adversity. Therefore, it is theoretically plausible that perceived minimization of health concerns may not directly relate to partners' resilience. Importantly, the health concern minimization subscale demonstrated significant associations with other theoretically relevant constructs (e.g., communication problems and FCR), supporting its convergent validity. Given the limited research in social constraints among partners of adults with cancer, further studies are needed to examine these relationships in diverse samples.

In line with a previous study in breast cancer patients (Cui et al., 2022), we found that educational level was the only demographic variable associated significantly with social constraints in partners of adults with cancer. Pairwise comparisons showed that the mean levels of social constraint in individuals with education levels of primary school or below were higher than those with education levels of junior secondary school, high secondary school, college, university or above. Interventions may target individuals with low education levels, especially those who completed primary school education or below. In China, low education often correlates with limited health literacy (Shan et al., 2023) and low income (Liu et al., 2025), exacerbating financial stress from cancer-related cost and restricting social participations. Healthcare providers can assess social constraint levels of partners of adults with cancer with the SC-SCS-P and provide timely and appropriate support.

### 4.1 Strengths and limitations

Our study has several strengths. Firstly, it was a multi-center study, recruiting participants in four study sites locating in central and eastern China. Furthermore, we included partners of adults newly diagnosed with cancer and partners of survivors capturing a broad span of the caregiving trajectory. These two sample characteristics enhanced the generalisability of the study findings. Secondly, the scale was adapted with a rigorous procedure, including expert panel validation and pilot testing, and was psychometrically tested in a large sample, thus enhancing the reliability and validity of the scale. However, the study had several limitations. The reliability of the validated scale for communication problem in the current study was questionable and might have impacted the validity of the findings. However, correlational findings of the SC-SCS-P with the other two related variables (psychological resilience and FCR) did provide evidence to support the convergent validity of the SC-SCS-P. Future studies are needed to explore other psychometric properties, such as the predictive validity of the scale. Besides, although five experts met the minimum acceptable standard, the relatively small panel size may limit the breadth of perspectives and could have contributed to the unanimous I-CVI results. Moreover, measurement invariance was not examined in the present study and should be tested in future research to further establish the structural stability of the instrument. Additionally, only 33 participants completed retest (21-day interval), representing 8.2% of the total sample. This small sample yields unstable ICC estimates. Future studies with larger retest samples (e.g., 50–100 participants) are recommended to obtain more stable reliability estimates. Furthermore, the present study focused exclusively on partners and did not collect patient-reported SCS data. Therefore, dyadic criterion validity could not be examined. Future dyadic research is warranted.

### 4.2 Implication for nursing research and practice

When people have social constraints, they encounter barriers in expressing their emotions and thoughts. For example, stopping talking about stressful experiences would keep individuals from confronting and processing stressful events, which might result in delayed or incomplete psychological adaptation (Zakowski et al., 2004). This scale may provide a useful screening measure to identify partners with a potentially social constraints condition and provide those affected partners with appropriate support, especially for those with low educational level. Specific recommendations could be provided for health care professionals on how to identify and address partners' social constraints. Some initial intervention strategies could be proposed to help reduce social constraints and its associated negative psychological impacts. For example, health care professionals can use the scale as a routine measure for partners of adults with cancer to assess their levels of social constraints for early detection and prevention. Tailored intervention for each of the three domains can also be developed to help partners with high social constraints score and use the scale as an outcome measure for interventional studies to reduce social constraints.

Both of the adults with cancer and their partners have social constraints as cancer diagnosis is a shared stressor. In order to prevent each other from being upset and burden, couples usually choose to hide or deny cancer-related concerns. Social constraints can affect their perception and disclosure of illness, coping behaviors, and psychological adjustment negatively (Lepore and Revenson, 2007; Cohee et al., 2017). Future studies can explore the level of social constraints and the associated factors between adults with cancer and their partners. Interventions can then be developed to help these families with social constraints by modifying the associated factors. From the homogeneity test in this study, partners with primary school or below tend to have higher social constraints level. Other study found cancer patients with a junior school or lower level of education were more likely to report a high level of social constraints (Cui et al., 2022). Therefore, interventions should be provided to those with low educational level. Furthermore, intimacy and relationship quality can be improved when one of the members discloses self-relevant feelings, and another member shows his/her responsiveness and feel understood. Therefore, interventions focusing on disclosure or communication between couples coping with cancer targeting on the core concept of social constraints are promising, which can simultaneously improve their psychological adjustment (Lepore and Revenson, 2007; Bu et al., 2025b). Given that social constraints was associated with psychological adjustment and communication, the reduction of social constraints is needed to help couples improve their psychological adjustment and potentially improve their relationship quality and intimacy by communication. Therefore, appropriate interventions that can reduce social constraints should be developed. Subsequently, improved outcomes for couples may be gained as well.

## 5 Conclusions

The adapted 15-item SC-SCS-P is a reliable and valid tool with strong evidence and can thus be used in measuring partner-specific social constraints from adults with cancer in China. The scale captures three key dimensions, including alienation, health concern minimization and emotional concealment. The development of the scale has filled the practice gap of no specific measurement of social constraints in partners of adults with cancer. This study lays the groundwork for research on social constraints among partners of adults with cancer and supports the design and evaluation of communication-focused interventions, with actionable implications for policy and practice.

## Data Availability

The data that support the findings of this study are available from the corresponding author upon reasonable request.

## References

[B1] Barrett P. (2007). Structural equation modelling: adjudging model fit. Pers. Individ. Dif. 42, 815–824. doi: 10.1016/j.paid.2006.09.018

[B2] Bian J. Zhang L. Liu Z. Ni T. Li Y. (2018). Reliability and validity of the revised Chinese version of benefit finding scale in family caregivers of cancer patients. Chin. Gen. Pract. 21, 2091–2096. doi: 10.3969/j.issn.1007-9572.2018.00.222

[B3] Boamah Mensah A. B. Adamu B. Mensah K. B. Dzomeku V. M. Agbadi P. Kusi G. . (2021). Exploring the social stressors and resources of husbands of women diagnosed with advanced breast cancer in their role as primary caregivers in Kumasi, Ghana. Support. Care Cancer 29, 2335–2345. doi: 10.1007/s00520-020-05716-2 32915296

[B4] Bray F. Laversanne M. Sung H. Ferlay J. Siegel R. L. Soerjomataram I. . (2024). Global cancer statistics 2022: GLOBOCAN estimates of incidence and mortality worldwide for 36 cancers in 185 countries. CA Cancer J. Clin. 74, 229–263. doi: 10.3322/caac.21834 38572751

[B5] Bu X. Chen X. Luo L. Fan R. Jiang L. Liu X. . (2025a). Preliminary testing for affiliate stigma scale: a reliable and valid stigma measure for caregivers of women with breast cancer. Asia Pac. J. Oncol. Nurs. 12:100652. doi: 10.1016/j.apjon.2024.100652 40026874 PMC11869986

[B6] Bu X. Jiang L. Leung D. Y. P. (2025b). Fear of cancer recurrence prevalence and its associated factors among family caregivers of women with breast cancer: a systematic review and meta-analysis. J. Clin. Nurs. 34, 2510–2524. doi: 10.1111/jocn.17680 39994915 PMC12181156

[B7] Campbell-Sills L. Stein M. B. (2007). Psychometric analysis and refinement of the connor-davidson resilience scale (CD-RISC): validation of a 10-item measure of resilience. J. Trauma. Stress 20, 1019–1028. doi: 10.1002/jts.20271 18157881

[B8] Cheng X. Du R. Zhou H. Zheng K. Chen C. Wang T. . (2021). Couple disease communication and its influencing factors among stoma patients with colorectal cancer. J. Nurs. Sci. 36, 10–13. doi: 10.3870/j.issn.1001-4152.2021.01.010

[B9] Cheung G. W. Cooper-Thomas H. D. Lau R. S. Wang L. C. (2024). Reporting reliability, convergent and discriminant validity with structural equation modeling: a review and best-practice recommendations. Asia Pac. J. Manag. 41, 745–783. doi: 10.1007/s10490-023-09871-y

[B10] Cohee A. A. Adams R. N. Johns S. A. Von Ah D. Zoppi K. Fife B. . (2017). Long-term fear of recurrence in young breast cancer survivors and partners. Psychooncology. 26, 22–28. doi: 10.1002/pon.4008 26490953 PMC4840075

[B11] Connor K. M. Davidson J. R. T. (2003). Development of a new resilience scale: the Connor-Davidson Resilience Scale (CD-RISC). Depress. Anxiety 18, 76–82. doi: 10.1002/da.10113 12964174

[B12] Cui C. Wang L. Wang X. (2022). Profiles of social constraints and associated factors among breast cancer patients: a latent profile analysis. BMC Psychiatry 22, 1–9. doi: 10.1186/s12888-022-04407-y 36451108 PMC9714186

[B13] Diao X. Guo C. Jin Y. Li B. Gao X. Du X. . (2025). Cancer situation in China: an analysis based on the global epidemiological data released in 2024. Cancer Commun. 45, 178–197. doi: 10.1002/cac2.12627 39659114 PMC11833671

[B14] e Silva T. B. Santos M. C. de Almeida A. M. Fernandes A. F. (2010). [The perception of mastectomized women's partners regarding life after surgery]. Rev. Esc. Enferm. USP 44, 113–119. doi: 10.1590/S0080-62342010000100016 20394227

[B15] Efthymiou A. Middleton N. Charalambous A. Papastavrou E. (2019). Adapting the eHealth literacy scale for carers of people with chronic diseases (eHeals-Carer) in a sample of Greek and Cypriot carers of people with dementia: reliability and validation study. J. Med. Internet Res. 21:e12504. doi: 10.2196/12504 31778120 PMC6908974

[B16] Fletcher D. Sarkar M. (2013). Psychological resilience: a review and critique of definitions, concepts, and theory. Eur. Psychol. 18, 12–23. doi: 10.1027/1016-9040/a000124

[B17] Gutierrez D. Barden S. M. Gonzalez J. Ali S. Cruz-Ortega L. G. (2016). Perspectiva masculina: an exploration of intimate partners of Latina breast cancer survivors. Fam. J. 24, 222–229. doi: 10.1177/1066480716648690

[B18] Heggestad E. D. Scheaf D. J. Banks G. C. Monroe Hausfeld M. Tonidandel S. Williams E. B. . (2019). Scale adaptation in organizational science research: a review and best-practice recommendations. J. Manage. 45, 2596–2627. doi: 10.1177/0149206319850280

[B19] Hendriks J. M. Crijns H. J. Tieleman R. G. Vrijhoef H. J. (2013). The atrial fibrillation knowledge scale: development, validation and results. Int. J. Cardiol. 168, 1422–1428. doi: 10.1016/j.ijcard.2012.12.047 23305860

[B20] Herrman H. Stewart D. E. Diaz-Granados N. Berger E. L. Jackson B. Yuen T. . (2011). What is resilience? Can. J. Psychiatry 56, 258–265. doi: 10.1177/070674371105600504 21586191

[B21] Hilton B. A. Crawford J. A. Tarko M. A. (2000). Men's perspectives on individual and family coping with their wives' breast cancer and chemotherapy. West. J. Nurs. Res. 22, 438–459. doi: 10.1177/019394590002200405 10826253

[B22] Ho D. Y.-f. (1976). On the concept of face. Am. J. Sociol. 81, 867–884. doi: 10.1086/226145

[B23] Hooper D. Coughlan J. Mullen M. R. (2008). Structural equation modelling: guidelines for determining model fit. Electron. J. Bus. Res. Methods 6, 53–60.

[B24] Hu L.-t. Bentler P. M. (2009). Cutoff criteria for fit indexes in covariance structure analysis: conventional criteria versus new alternatives. Struct. Equ. Model. 6, 1–55. doi: 10.1080/10705519909540118

[B25] Just Enough R. (n.d.). Modification Indices. Available online at: https://benwhalley.github.io/just-enough-r/modification-indices.html (Accessed January 2, 2026).

[B26] Kane R. L. (2006). Understanding Health Care Outcomes Research, 2nd edn. Sudbury, MA: Jones and Bartlett.

[B27] Kim H. Y. (2013). Statistical notes for clinical researchers: assessing normal distribution using skewness and kurtosis. Restor. Dent. Endod. 38, 52–54. doi: 10.5395/rde.2013.38.1.52 23495371 PMC3591587

[B28] Koo T. K. Li M. Y. (2016). A guideline of selecting and reporting intraclass correlation coefficients for reliability research. J. Chiropr. Med. 15, 155–163. doi: 10.1016/j.jcm.2016.02.012 27330520 PMC4913118

[B29] Kornblith A. B. Regan M. M. Kim Y. Greer G. Parker B. Bennett S. . (2006). Cancer-related communication between female patients and male partners scale: a pilot study. Psychooncology 15, 780–794. doi: 10.1002/pon.1004 16308887

[B30] Koutrouli N. Anagnostopoulos F. Tsikkinis A. Papastylianou D. Lepore S. (2016). Psychometric properties of the Greek version of the Social Constraints Scale in a sample of women with breast cancer. Women Health 56, 413–427. doi: 10.1080/03630242.2015.1101746 26496047

[B31] Kyriazos T. (2018). Applied psychometrics: sample size and sample power considerations in factor analysis (EFA, CFA) and SEM in general. Psychology 9, 2207–2223. doi: 10.4236/psych.2018.98126

[B32] Lepore S. J. Revenson T. A. (2007). Social constraints on disclosure and adjustment to cancer. Soc. Personal. Psychol. Compass 1, 313–333. doi: 10.1111/j.1751-9004.2007.00013.x

[B33] Lepore S. J. Silver R. C. Wortman C. B. Wayment H. A. (1996). Social constraints, intrusive thoughts, and depressive symptoms among bereaved mothers. J. Pers. Soc. Psychol. 70, 271–282. doi: 10.1037/0022-3514.70.2.271 8636882

[B34] Liu Y. Li P. Liu B. Qi Y. Guan W. Zhang N. . (2025). Effect of socioeconomic status on financial toxicity: the chain mediating roles of social support and self-efficacy. Cancer Med. 14:e71083. doi: 10.1002/cam4.71083 40709597 PMC12290632

[B35] Luo Z. Wang H. Hua L. Zhou L. Cheng T. Cheng H. . (2023). Cross-cultural adaptation and validation of the 15-item Social Constraint Scale (Cancer-spouse Version) in cancer patients. J. Nurs. Sci. 38, 40–43. doi: 10.3870/j.issn.1001-4152.2023.24.040

[B36] MacCallum R. C. Browne M. W. Sugawara H. M. (1996). Power analysis and determination of sample size for covariance structure modeling. Psychol. Methods 1, 130–149. doi: 10.1037/1082-989X.1.2.130

[B37] Marín-Maicas P. Portillo M. C. Corchón S. Ambrosio L. (2024). Methodological proposal for the adaptation of the living with long-term conditions scale to the family caregiver. Nurs. Rep. 14, 532–544. doi: 10.3390/nursrep14010041 38535713 PMC10974583

[B38] Mehnert A. Herschbach P. Berg P. Henrich G. Koch U. (2006). [Fear of progression in breast cancer patients–validation of the short form of the Fear of Progression Questionnaire (FoP-Q-SF)]. Z. Psychosom. Med. Psychother. 52, 274–288. doi: 10.13109/zptm.2006.52.3.274 17156600

[B39] Nasiri A. Taleghani F. Irajpour A. (2016). Adjustment process in Iranian men to their wives' breast cancer. Eur. J. Cancer Care. 25, 307–317. doi: 10.1111/ecc.12293 25684401

[B40] Polit D. F. Beck C. T. Owen S. V. (2007). Is the CVI an acceptable indicator of content validity? Appraisal and recommendations. Res. Nurs. Health 30, 459–467. doi: 10.1002/nur.20199 17654487

[B41] Shan Y. Ji M. Xing Z. Dong Z. (2023). Factors associated with limited cancer health literacy among Chinese people: cross-sectional survey study. JMIR Form. Res. 7:e42666. doi: 10.2196/42666 37223982 PMC10248776

[B42] Simonelli L. E. Siegel S. D. Duffy N. M. (2017). Fear of cancer recurrence: a theoretical review and its relevance for clinical presentation and management. Psychooncology 26, 1444–1454. doi: 10.1002/pon.4168 27246348

[B43] Soriano E. C. Otto A. K. LoSavio S. T. Perndorfer C. Siegel S. D. Laurenceau J. P. (2021). Fear of cancer recurrence and inhibited disclosure: testing the social-cognitive processing model in couples coping with breast cancer. Ann. Behav. Med. 55, 192–202. doi: 10.1093/abm/kaaa043 32608472 PMC7980765

[B44] Suan M. A. M. Tan W. L. Soelar S. A. Ali A. M. (2017). The development and validation of the nurses' attitude towards conducting research questionnaire (NA 2 CRESQ). Ann. Med. Health Sci. Res. 7, 377–382.

[B45] Tavakol M. Wetzel A. (2020). Factor analysis: a means for theory and instrument development in support of construct validity. Int. J. Med. Educ. 11, 245–247. doi: 10.5116/ijme.5f96.0f4a 33170146 PMC7883798

[B46] Traboulssi M. Pidgeon M. Weathers E. (2022). My wife has breast cancer: the lived experience of Arab men. Semin. Oncol. Nurs. 38:151307. doi: 10.1016/j.soncn.2022.151307 35688767

[B47] Wang L. Geng X. Ji L. Lu G. Lu Q. (2020). Treatment decision-making, family influences, and cultural influences of Chinese breast cancer survivors: a qualitative study using an expressive writing method. Support. Care Cancer 28, 3259–3266. doi: 10.1007/s00520-019-05161-w 31735999

[B48] Wang L. Shi Z. Zhang Y. Zhang Z. (2010). Psychometric properties of the 10-item Connor-Davidson Resilience Scale in Chinese earthquake victims. Psychiatry Clin. Neurosci. 64, 499–504. doi: 10.1111/j.1440-1819.2010.02130.x 20923429

[B49] Williams B. Onsman A. Brown T. (2010). Exploratory factor analysis: a five-step guide for novices. Australas. J. Paramed. 8, 1–13. doi: 10.33151/ajp.8.3.93

[B50] Wolf R. (1967). Evaluation of several formulae for correction of item-total correlations in item analysis. J. Educ. Measure. 4, 21–26. doi: 10.1111/j.1745-3984.1967.tb00565.x

[B51] Wu Q. (2016). The status research and study on fear of progression of primary liver cancer patients and their spouses Master's thesis, Naval Medical University.

[B52] Yang X. Li H. Zhang J. Li P. Li M. Hao T. . (2022). Status of cancer-related communication problems from the perspective of spouses of young and middle-aged breast cancer patients. J. Nurs. Sci. 37, 51–55. doi: 10.3870/j.issn.1001-4152.2022.24

[B53] Yeung N. C. Y. Ramirez J. Lu Q. (2017). Perceived stress as a mediator between social constraints and sleep quality among Chinese American breast cancer survivors. Support. Care Cancer 25, 2249–2257. doi: 10.1007/s00520-017-3632-9 28190157

[B54] Yoshimochi L. T. B. Santos M. A. D. Loyola E. A. C. Magalhães P. A. P. Panobianco M. S. (2018). The experience of the partners of women with breast cancer. Rev. Esc. Enferm. USP. 52:e03366. doi: 10.1590/s1980-220x2017025203366 30403266

[B55] Zahlis E. H. Lewis F. M. (2010). Coming to grips with breast cancer: the spouse's experience with his wife's first six months. J. Psychosoc. Oncol. 28, 79–97. doi: 10.1080/07347330903438974 20391067 PMC2856107

[B56] Zahlis E. H. Shands M. E. (1991). Breast cancer: demands of the illness on the patient's partner. J. Psychosoc. Oncol. 9, 75–93. doi: 10.1300/J077v09n01_04

[B57] Zakowski S. G. Ramati A. Morton C. Johnson P. Flanigan R. (2004). Written emotional disclosure buffers the effects of social constraints on distress among cancer patients. Health Psychol. 23, 555–563. doi: 10.1037/0278-6133.23.6.555 15546223

[B58] Zhang B. Xiao Q. Gu J. Ma Q. Han L. (2024). A qualitative study on the disease coping experiences of pancreatic cancer patients and their spouses. Sci. Rep. 14:18626. doi: 10.1038/s41598-024-69599-7 39128911 PMC11317503

